# Epidemiology of tuberculosis in a low-incidence Italian region with high immigration rates: differences between not Italy-born and Italy-born TB cases

**DOI:** 10.1186/1471-2458-11-376

**Published:** 2011-05-23

**Authors:** Anna Odone, Matteo Riccò, Matteo Morandi, Bianca M Borrini, Cesira Pasquarella, Carlo Signorelli

**Affiliations:** 1 Department of Public Health, University of Parma, Parma, Italy; 2 Agenzia Sanitaria e Sociale Regionale Emilia-Romagna, Area di Programma Rischio Infettivo, Bologna, Italy; 3 Servizio Sanità Pubblica - Direzione Generale Sanità e Politiche Sociali - Regione Emilia-Romagna, Italy

## Abstract

**Background:**

Emilia Romagna, a northern Italian region, has a population of 4.27 million, of which 9.7% are immigrants. The objective of this study was to investigate the epidemiology of tuberculosis (TB) during the period 1996-2006 in not Italy-born compared to Italy-born cases.

**Methods:**

Data was obtained from the Regional TB surveillance system, from where personal data, clinical features and risk factors of all notified TB cases were extracted.

**Results:**

5377 TB cases were reported. The proportion of immigrants with TB, over the total number of TB cases had progressively increased over the years, from 19.1% to 53.3%. In the not Italy-born population, TB incidence was higher than in Italians (in 2006: 100.7 cases per 100 000 registered not Italy-born subjects and 83.9/100 000 adding 20% of estimated irregular presences to the denominators. TB incidence among Italians was 6.5/100 000 Italians). A progressive rise in the not Italy-born incident cases was observed but associated with a decline in TB incidence. Not Italy-born cases were younger compared to the Italy-born cases, and more frequently classified as "new cases" (OR 2.0 95%CI 1.61-2.49 for age group 20-39); 60.7% had pulmonary TB, 31.6% extra pulmonary and 7.6% disseminated TB. Risk factors for TB in this population group were connected to lower income status (homeless: OR 149.9 95%CI 20.7-1083.3 for age group 40-59).

**Conclusions:**

In low-incidence regions, prevention and control of TB among sub-groups at risk such as the foreign-born population is a matter of public health concern. In addition, increasing immigration rates may affect TB epidemiology. TB among immigrants is characterized by particular clinical features and risk factors, which should be analyzed in order to plan effective action.

## Background

With 9.4 million new cases estimated in 2009 by the World Health Organization (WHO), tuberculosis (TB) is a worldwide epidemic [1]. The regions most affected by TB are in low-income and middle-income countries within Asia and sub-Saharan Africa. In industrialised countries, even tough a decline in TB incidence rates has been shown over the last century [2], an increase in immigration [3] of people from high TB incidence areas has recently contributed to reverse this downward trend [4-6] and to the re-emergence of TB as a matter of public health concern [2,7].

In the United States and in Western European countries, the proportion of TB cases in immigrants of the total prevalence rates increases every year [8-10]. In 2006, the percentage of foreign-born subjects with TB in Norway was 81% [9] of the total prevalence, in Sweden 71.8% [9] and 57.3% in Denmark [9]. Other European countries such as Belgium [9], The Netherlands [9], Switzerland [9,11] and The United Kingdom [9] experienced figures over 50%.

Italy, in line with this trend, has witnessed an increase in the not Italy-born population by two-fold, over the last four years [12]. In addition, the proportion of immigrants with TB over the total number of TB cases notified in this country reached 46.2% in 2006 [13].

Aim of the current study was to describe the occurrence of TB in the northern Italian region Emilia Romagna over an eleven-year period (1996-2006) and to explore its determinants in two different populations: the not Italy-born subjects and the Italy-born ones. In particular, the differences between not Italy-born and Italy-born TB cases concerning personal data, clinical features and risk factors for TB infection and transmission were analyzed.

## Methods

### Setting

Emilia Romagna, has 4.27 million inhabitants, is an administrative region in the north of Italy and the third richest in the country. According to the Italian National Institute of Statistics, the proportion of resident immigrants (subjects who own an official residence permit) relative to the whole population has increased from 1.1% in 1993 to 9.7% in 2008 [12], which is now the highest in Italy.

Quantify the presence of not Italy-born subjects in Italy is hard. We considered data of the Italian National Institute of Statistics on country of birth and residence permits to differentiate between "not Italy-born" and "Italy-born" subject and obtain an estimation of their presence. In addition, not Italy-born subjects were differentiated in "regularly registered" and "not regularly registered" depending on possession of residence permit.

### Background

The Italian Healthcare system provides health care to all acute patients regardless of nationality or legal and insurance status.

Since 1934, TB notification has been mandatory in Italy and a system of surveillance that follows a vertical flow from the physician's notification to the Local Health Authority, then to the Regional Authority and from here to the Ministry of Health and to the WHO has been implemented by national decrees. (Ministry of Health, Ministerial Decree of 15^th ^December 1990 and Ministerial Decree of 29^th ^July 1998).

### Data sources and statistical analysis

Data were obtained from the Regional TB surveillance system active in Emilia Romagna from 1996 onwards. It compiles TB notifications of people diagnosed with TB in the region, both residents and not residents, Italy-born or not Italy-born. It includes also TB notifications of people without a legal residence permit. It regularly records surveillance data, data on treatment outcome and from 2005 onwards, also data on drug resistances.

From the database of the Regional TB surveillance system we extracted relevant information to describe characteristics of TB-affected population, to identify specific sub-groups at risk and to analyze temporal trend of disease in the region over the years. These included personal data counting age, sex, place of birth and of residence of TB cases. In addiction, we extracted TB disease characteristics for each case such as clinical and microbiological features, diagnostic criteria, TB type ("new case", where a first time TB diagnosis is made and reported, or "re-treated cases"), site of disease (pulmonary, extra pulmonary or disseminated) and risk factors for TB; both behavioural risk factors, contact history; alcoholism; drug addiction and clinical risk factors, chronic and degenerative disease; disorders of the immune system.

Number of TB cases and TB incidence (/100 000 population per year) were investigated for the considered period both for the total population and for the two principal subgroups: the Italy-born and the not Italy-born population. Annual trends for number of TB cases and TB incidence for Italy-born and not Italy-born subjects were investigated through a linear regression model and estimate of unstandardized coefficients (B) and their confidential intervals (95%CI). In addition, a p value labelling the observed significance level for the t statistics (which tested the hypothesis that B was 0) was reported.

Calculation of TB incidence in the not Italy-born population was made with two different methods: first considering as denominators the data on immigrants regularly registered (subjects who own a regular residence permit) in Emilia-Romagna and secondly adding to these data the estimated prevalence of not regularly registered immigrant presence. The demographic data to derive population denominators and calculate TB incidence were obtained from the public-use files of the Italian National Institute of Statistics and refers to the balances on resident population on 31st December of the previous year for each considered year [11]. For the not Italy-born population, data on resident population are only available from year 2002 [12]. Regarding the estimation of not regularly registered immigrants, it was considered to be 20% of regular immigrants for every year, excluding 2003 the year that followed after the approval of a national decree which massively legitimized irregular status, where it was considered to be 10%. This estimation of not regularly registered immigrant presence had been previously inferred in the section on foreigners' health status in the 2007 report of the National Observatory of health in Italian Regions, a highly qualified Italian scientific institution [14].

Study variables were analyzed differentiating between Italy-born and not Italy-born cases. Comparison between groups were made using the calculation of odds ratio (OR) and its 95% confidential intervals (95%CI). Continuous variables (i.e. age at notification) were compared using the Student t-test for unpaired data.

Statistical analysis was performed using the Statistical Package for Social Sciences, for MacOsX, version 16.01 (SPSS Inc. Chicago, IL, USA).

### Ethics

The protocol for this study was reviewed and granted by the Institutional Board of the Department of Public Health at the University of Parma and by the Regional Health Authorities and found to be public health surveillance and not human subject research.

## Results

The study included 5377 TB cases notified from 1996 through 2006: 3148 (58.5%) males and 2229 (41.5%) females. Of the total, 1964 (36.5%) were not Italy-born, while 3413 (63.4%) were Italy-born. Table 1 shows the geographical origins of not Italy-born TB cases; Morocco, the principal country of origin of immigrants in Emilia-Romagna (53571 registered subjects in 2006 [12]), was the most represented country among not Italy-born TB cases (28.3%, n = 398 cases).

**Table 1:** Geographical origins of not Italy-born TB cases by World Health Organization (WHO) region

WHO Region	Number of TB cases	% of not Italy-born TB cases
Eastern Mediterranean WHO region (WHO-EMRO)	719	36.6%

The African region (WHO-AFRO)	425	21.7%

European region (WHO-EURO)	386	19.7%

South East Asia region (WHO-SEARO)	225	11.5%

American region (WHO-AMRO)	106	5.4%

Western Pacific region (WHO-WPRO)	95	4.8%

**Figure 1:**
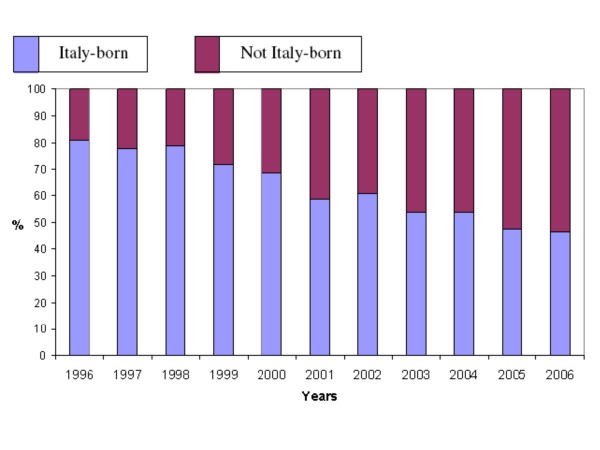
Proportion of Italy-born and not Italy-born subjects among TB cases, Emilia-Romagna region, Italy: 1996-2006

Total TB cases were, on average, 488.8 per year and no significant time trends were found. Not Italy-born TB cases showed an increase of 213% during the study period (from 93 in 1996 to 291 in 2006). The annual proportion of not Italy-born cases on the total TB notifications increased from 19.1% in 1996, to 53.3% in 2006 (Figure 1). A linear regression analysis of the data confirmed a statistically significant increasing trend in the number of not Italy-born cases (B = 19.53; 95%CI 17.19 - 21.88; p < 0.0001) and a concurrently statistically significant decreasing trend in the notification rate among Italy-born people (B = -16,36; 95%CI -21.26 - -11.47; p < 0.0001).

The TB incidence for the whole population varied between 12.5 new cases/100 000 inhabitants in 1996 and 13/100 000 in 2006, this increase being not statistically significant (B = -0.01; 95%CI -0.14 - 0.12; p = 0.8107). Data were then segregated by geographical origin of subjects showing higher rates for not Italy-born subject as compared to Italy-born ones. In fact, for Italy-born TB cases, TB incidence decreased from 7.6/100 000 in 2002 to 6.5/100 000 in 2006 (decreasing trend not statistically significant; B = -0.39; 95%CI -0.94 - 0.16; p = 0.1090). In the not Italy-born population with regular permit of residence, TB incidence varied from 141.4/100 000 in 2002 to 100.7/100 000 in 2006. In this population the previously mentioned rise in number of TB cases observed over the considered time-frame, was not associated with increasing incidence trend. In fact, a statistically significant decrease in TB incidence was revealed among not Italy-born subjects (B = -12.51; 95%CI -22.94 - -2.09; p = 0.0316). This pattern suggests that the increase of total TB cases over the study period is only due to higher immigration rates.

**Figure 2:**
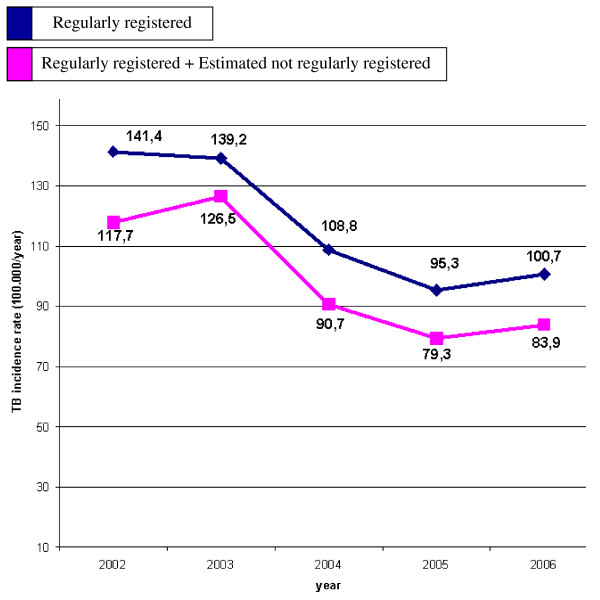
**TB incidence rates (/100.000 population per year) in the not Italy-born population: denominators with and without estimated not regularly registered presences**. Emilia Romagna region, Italy: 1996-2006.

Figure 2 shows two different estimations for TB incidence among the not Italy-born population: first considering only legally registered immigrants in the denominator and secondly adding an inferred percentage of not regularly registered immigrants. In 2006, TB incidence was calculated to be 100.7/100 000 registered not Italy-born subjects, and 83.9/100 000 when an estimated figure of 20% not regularly registered presences was included.

The mean age for Italy-born people diagnosed with TB was 62.1 years (±11.5 SD), while not Italy-born cases were significantly younger (mean age of 32.8 ± 20.1 years; p value of Student's t-test for unpaired data <0.0001). 84.8% of the not Italy-born subjects were between 15 and 44 years old.

Classification of TB type (Table 2) at the moment of diagnosis was available for 2 897 patients (233 not Italy-born subjects and 1 664 Italians), 53.9% of the total. A total of 2 606 (89.9%) were classified as "new cases", 291 (10.1%) as "re-treated cases", either failure or relapses. Not Italy-born TB patients were more frequently "new cases", and were more prominent in the 20 to 39 year old age group. (OR 2.0 95%CI 1.61-2.49). The "unknown" TB type status was significantly associated with young not Italy-born TB patients.

**Table 2:** Odd ratios for type of disease according to origin of patients (not Italy-born/Italy-born) by age group

	New case			Re-treatment case			Unknown		
	
Age group (years)	Not Italy-b n	Italy-b^ n	OR 95% CI	Not Italy-b n	Italy-b^ n	OR 95% CI	Not Italy-b n	Italy-b^ n	OR 95% CI
**0 - 19**	85	73	0.96 (0.59 - 1.58)	2	1	1.70 (0.15 - 19.0)	26	8	3.14 (1.36 - 7.22)**
**20 - 39**	820	187	2.00 (1.61 - 2.49)***	43	17	0.80 (0.45 - 1.42)	187	19	3.49 (2.15 - 5.67)***
**40 - 59**	230	290	1.85 (1.43 - 2.38)***	18	30	1.02 (0.56 - 1.86)	65	44	2.81 (1.88 - 4.22)***
**60 - 79**	25	650	1.37 (0.79 - 2.40)	7	132	1.74 (0.77 - 3.95)	7	108	2.17 (0.95 - 4.92)
**≥80**	2	244	0.99 (0.16 - 5.99)	1	40	3.55 (0.39 - 32.5)	1	67	2.02 (0.22 - 18.3)
**Total**	1162	1444	1.98 (1.76 - 2.21)***	71	220	1.98 (1.76 - 2.21)***	282	246	2.19 (1.83 - 2.63)***

* = p < 0.05; ** = p < 0.01; *** = p < 0.001

^Reference category

Detection methods, 3 224 (60%) of the subjects presented clinical symptoms suggesting TB. Concerning pulmonary TB cases, a chest X-ray was performed on 3243 (96.5%) subjects (2589 positive, 654 negative), smear on 1666 (49.6%) subjects (689 positive, 977 negative). Overall the tuberculin test was performed on 2415 (45%) subjects (1849 positive, 566 negative), culture on 4560 (84.8%) subjects (3418 positive, 1142 negative) and histology on biopsy specimen on 1730 (32%) subjects (691 positive, 1039 negative). Not Italy-born subjects were more likely identified by a positive chest X-ray (OR 2.14 95%CI 1.91-2.39), by a positive tuberculin test (OR 2.44 95%CI 2.11-2.83), or by clinical TB symptoms (OR 2.74 95%CI 2.42-3.09). Not Italy-born subjects showed a higher positivity both to sputum smear examination (OR 1.53 95%CI 1.33-1.76) and to cultural examination (OR 1.14 95%CI 1.00-1.31).

Concerning TB localization (Table 3), 62.4% (n = 3358) had pulmonary TB and 31.2% (n = 1679) extra pulmonary TB. 6% (n = 324) were disseminated TB forms. Common extra pulmonary sites included the peripheral lymph nodes, the genitourinary tract and the pleura, which were affected in 10.6%, 5% and 4.5% of all tuberculosis cases reported, respectively. Less frequent incidents were gastro-intestinal (1.7%), vertebral (1.4%), osseous non-vertebral (1.2%) and meningeal (0.5%) localization. The likelihood of disseminated TB was significantly higher among immigrants (age group 40 to 59 years: OR 1.82 95%CI 1.08-3.06). Extra pulmonary disease was slightly more common among not Italy-born subjects, although this difference was not statistically significant in all age groups.

**Table 3:** Odd ratios for site of disease according to origin of patients (not Italy-born/Italy-born) by age group

	Pulmonary TB			Extra-pulmonary TB			Disseminated TB		
	
Age group (years)	Not italy-b n	Italy-b^ n	OR 95% CI	Not italy-b n	Italy-b^ n	OR 95% CI	Not italy-b n	Italy-b^ n	OR 95% CI
**0 - 19**	81	79	0.69 (0.42 - 1.14)	52	36	1.35 (0.80 - 2.26)	10	6	1.44 (0.51 - 4.09)

**20 - 39**	846	305	0.71 (0.56 - 0.89)**	419	111	1.30 (1.02 - 1.66)*	106	21	1.67 (1.03 - 2.70)*

**40 - 59**	228	422	0.80 (0.62 - 1.04)	132	212	1.09 (0.83 - 1.42)	31	30	1.82 (1.08 - 3.06)*

**60 - 79**	31	960	0.99 (0.56 - 1.77)	17	533	0.98 (0.54 - 1.77)	3	81	1.16 (0.35 - 3.79)

**≥80**	5	401	1.52 (1.43 - 1.60)	0	167	-	0	37	-

**Total**	1191	2167	0.89 (0.79 - 0.99)*	620	1059	1.02 (0.91 - 1.16)	150	175	1.53 (1.22 - 1.91)***

* = p < 0.05; ** = p < 0.01; *** = p < 0.001

^ Reference category

The risk factors for TB identified in our study are presented in Table 4, stratified by age groups. About half of the subjects had identifiable risk factors. Homeless status (OR 149.9 95%CI 20.7-1083.3, for age group 40 to 59 years) was more frequently associated with young not Italy-born patients as well as prisoner status, the latter being not significant. Not Italy-born subjects were more frequently subject to household contact being adults, while at a younger age (0 to 19 years) this condition was significantly associated to Italian TB cases (OR 0.26 95%CI 0.13-0.50). Among behavioural risk factors, there were no significant differences concerning alcoholism while *i.v. *drug use appeared to be significantly linked to young Italians. Moreover, HIV infection in young people resulted to be a risk factor for TB especially for Italians (20 to 39 years: OR 0.35 95%CI 0.24-0.5). Compared to Italian-born subjects, not Italy-born subjects rarely had a history of cancer at the moment of TB diagnosis while in age group 60 to 79 years, they had significant likelihood of having diabetes (OR 2.53 95%CI 1.16-5.52).

**Table 4:** Odd ratios for TB risk factors according to origin of patients (not Italy-born/Italy-born) by age group

Risk factor	n. not Italy-born cases + n. Italy-born cases^ Odds Ratio (95% CI)				
	**0 - 19 y** **(n = 143 + 121)**	**20 - 39 y** **(n = 1374 + 440)**	**40 - 59 y** **(n = 391 + 665)**	**60 - 79 y** **(n = 51 + 1579)**	**≥80 y** **(n = 5 + 608)**

Homeless (n = 416)	39 + 2 22.3 (5.26 - 94.66)***	292 + 0 -	72 + 1 149.9 (20.7 - 1083.3)***	9 + 0 -	1 + 0 -

Prison (n = 30)	0 + 0 -	18 + 3 1.93 (0.57 - 6.60)	0 + 7 -	0 + 2 -	0 + 0 -

Case contact (n = 150)	14 + 36 0.26 (0.13 - 0.50)***	82 + 33 0.79 (0.52 - 1.19)	16 + 27 1.01 (0.54 - 1.90)	0 + 37 -	0 + 5 -

HIV+ (n = 180)	0 + 0 -	66 + 56 0.35 (0.24 - 0.50)***	15 + 27 0.94 (0.50 - 1.79)	0 + 12 -	0 + 4 -

Alcohol (n = 63)	0 + 0 -	18 + 2 2.91 (0.67 - 12.58)	8 + 18 0.75 (0.32 - 1.74)	0 + 16 -	0 + 1 -

*i.v*. Drug user (n = 47)	1 + 0 -	19 + 17 0.35 (0.18 - 0.68)**	0 + 7 -	0 + 3 -	0 + 0 -

Diabetes (n = 231)	0 + 0 -	9 + 2 1.44 (0.31 - 6.71)	23 + 37 1.06 (0.62 - 1.81)	8 + 108 2.53 (1.16 - 5.52)*	0 + 44 -

Disorders of absorption (n = 123)	4 + 1 3.45 (0.39 - 31.32)	21 + 6 1.12 (0.45 - 2.80)	9 + 10 1.54 (0.62 - 3.83)	3 + 42 2.29 (0.68 - 7.64)	0 + 27 -

Chronic Renal Failure (n = 64)	0 + 0 -	5 + 1 1.60 (0.19 - 13.76)	5 + 6 1.42 (0.43 - 4.69)	0 + 33 -	0 + 12 -

Cancer (n = 305)	1 + 2 0.42 (0.04 - 4.68)	4 + 7 0.18 (0.05 - 0.62)**	5 + 34 0.24 (0.09 - 0.62)**	0 + 195 -	0 + 57 -

Working as healthcare professional (n = 56)	0 + 0 -	6 + 23 0.08 (0.03 - 0.20)***	0 + 18 -	0 + 7 -	0 + 2 -

* = p < 0.05; ** = p < 0.01; *** = p < 0.001

^ Reference category

## Discussion

The TB notifications in the Emilia Romagna region number about 12.1% of all Italian notifications. Regional TB incidence estimation is similar to other northern and central region realities [15-17] and are higher comparable to national data (in 2006: 12.5 cases per 100 000 population in Emilia Romagna and 7.7 in Italy [13]). In developed countries like Italy, TB has increasingly become a disease of specific population subgroups; immigrants are a population group at risk and their presence may affect the TB epidemiologic situation in host countries [5,6,18].

In this study, which considers an 11-year period, a reduction in the proportion of Italy-born TB cases and a corresponding rise in the not Italy-born cases were observed. In addition, the increase of not Italy-born TB cases resulted to be combined with progressively increased immigration in the region as TB incidence has shown a progressive reduction. These findings are in accordance with trends at the national level [13,14].

Limitations in estimating TB incidence must be mentioned. Denominators for the immigrant population which only considers documented not Italy-born subjects in Emilia Romagna (not including immigrants without a legal residence permit) leads to overestimation of TB incidence. Not regularly registered immigrants are not easily quantified and no official data are available in the Emilia-Romagna region. The estimate of 20% (10% in 2003) of not regularly registered status taken into account is approximate. In addition, differentiating between Italy-born and not Italy-born cases doesn't allow including young foreigners who were born in Italy in the immigrant sub group; this being another limiting aspect. However, it is realistic to consider that the TB incidence in the not Italy-born population is much higher than in the Italy-born population but it doesn't tend to increase over time as understood from our and other studies [13,14]. The present study being based on notified cases, as a unique source of information is another limiting aspect. In fact, notification data, although the surveillance system is considered quite efficient in Italy, can be hampered by under-reporting and inconsistency. In addition, even if healthcare assistance is offered also to "not regularly registered" immigrants, not Italy-born subjects without legal residence permit might be reluctant to seek healthcare, this attitude leading to underestimation of TB cases and being a matter of public health concern [19].

Taking into consideration the massive and constantly increased immigration in Italy, and the implication on TB epidemiology, it is of utmost importance to differentiate tubercular disease among not Italy-born subjects. In our study, as reported in other developed areas [20-24], TB among immigrants is mostly found in young adults, while in the native population, TB is mostly diagnosed at an older age. This could be partially linked to the immigration of predominantly young people but it also reflects true epidemiologic differences in TB patterns of transmission in low-incidence countries like Italy and intermediate and high-incidence countries like countries of origin of immigrants. In this context, it is more complex to demonstrate whether TB in immigrants is a consequence of an infection acquired in the country of origin [22,25] or in the host country [26,27] as specific molecular investigations are needed but were not available for this study.

The higher risk showed by immigrants of being "new cases" rather than "re-treated cases" might be linked both to the natural history of infection and to their younger age. Poor past medical documentation and communication difficulties during medical examinations may be a contributing factor in hiding previous TB disease or treatments and could explain why the majority of immigrants are classified as "new cases" or "unknown" as the information is missing. TB diagnosis in older Italy-born patients, most frequently diagnosed as "re-treatment cases", probably reflects reactivation of old infections [28].

Differentiating between different risk factors for TB and evaluating their importance both for single individuals and for specific subgroups is a fundamental step for containing infection, informing preventive policies and designing effective TB control measures. The present study detected a major difference between the Italy-born and the not Italy-born subjects with reference to risk factors. Firstly, coming from a high-TB-incidence country is a major immigrant population risk factor [29]. Contrary to a supposed higher association with HIV infection in TB patients coming from countries with high prevalence of HIV infection, stratifying by age demonstrated young Italians to be highly associated with HIV infection and drug abuse, confirming them to be a subgroup at risk. Immigration conditions in the host country is often indicative of living in crowded places, being homeless or in prison, all well-known risk factors for TB infection, reactivation, transmission and TB disease progression [20,30,31].

Relating to TB disease sites, differences in the likelihood of extra pulmonary TB depending on immigration status remain largely unclear [32]. Data in our possession do not show a significant higher risk of extra pulmonary TB for immigrants. One aspect for concern is that extra pulmonary TB forms are more likely to be under-reported or not suspected by health specialists often inadequately trained in TB diagnosis.

## Conclusions

TB in the immigrant population is a major public health problem in Italy as well as in other low-incidence countries. Although it cannot be precisely quantified, TB incidence in not Italy-born nationals is higher than in the native population. TB surveillance among this specific at-risk population should be made a priority as immigration flow to Italy is increasing at a fast rate.

This study aimed at analysing how TB epidemiology in Emilia Romagna has been affected by an increase in the immigrant population and to differentiate between not Italy-born subjects and Italy-born subjects in terms of demographical and social data, specific risk factors, and clinical features. This knowledge might be of considerable help to plan tailored and effective prevention campaigns and surveillance programmes both in the Emilia Romagna region and at a national Italian level as well as in other TB low-incidence areas with similar immigration flows, socio-economic situation and health policy characteristics.

## Competing interests

The authors declare that they have no competing interests.

## Authors' contributions

AO participated in the design of the study, as well as the acquisition, documentation, analysis and interpretation of data, and the drafting of the article. MR participated in conception and design and in the analysis and interpretation of data. MM and BMB have contributed to conception and design and acquisition of data. CP and CS participated in the coordination, the critical revision of the study, for important intellectual content and gave final approval of the version to be published. All authors read and approved the manuscript.

## Pre-publication history

The pre-publication history for this paper can be accessed here:

http://www.biomedcentral.com/1471-2458/11/376/prepub
